# ﻿Morphology, phylogeny and host specificity of two new *Ophiocordyceps* species belonging to the “zombie-ant fungi” clade (Ophiocordycipitaceae, Hypocreales)

**DOI:** 10.3897/mycokeys.99.107565

**Published:** 2023-10-16

**Authors:** Dexiang Tang, Jing Zhao, Yingling Lu, Zhiqin Wang, Tao Sun, Zuoheng Liu, Hong Yu

**Affiliations:** 1 Yunnan Herbal Laboratory, College of Ecology and Environmental Sciences, Yunnan University, Kunming, 650504, China Yunnan University Kunming China; 2 School of Life Science, Yunnan University, Kunming, 650504, China Yunnan University Kunming China

**Keywords:** *Colobopsis*, Entomopathogenic fungi, *Ophiocordyceps*, Taxonomy

## Abstract

Species of the genus *Ophiocordyceps*, which include species able to manipulate the behaviour of ants, are known as the “zombie-ant fungi” and have attracted much attention over the last decade. They are widespread within tropical, subtropical and even temperate forests worldwide, with relatively few reports from subtropical monsoon evergreen broad-leaved forest. Fungal specimens have been collected from China, occurring on ants and producing hirsutella-like anamorphs. Based on a combination of morphological characters, phylogenetic analyses (LSU, SSU, *TEF1a*, *RPB1* and *RPB2*) and ecological data, two new species, *Ophiocordycepstortuosa* and *O.ansiformis*, are identified and proposed herein. *Ophiocordycepstortuosa* and *O.ansiformis* are recorded on the same species of *Colobopsis* ant, based on phylogenetic analyses (*COI*), which may be sharing the same host. *Ophiocordycepstortuosa* and *O.ansiformis* share the morphological character of producing lanceolate ascospores. They have typical characteristics distinguished from other species. The ascospore of *O.tortuosa* are tortuously arranged in the ascus and the ascospore of *O.ansiformis* have a structure like a handle-shape in the middle. Our molecular data also indicate that *O.tortuosa* and *O.ansiformis* are clearly distinct from other species.

## ﻿Introduction

Fungi associated with insects, morphologically similar, but genetically distinct cryptic closely-related species, have given rise to spectacular diversity across a wide range of taxa in the kingdom of fungi. Molecular studies have routinely unmasked several cryptic species and have revealed this as a common phenomenon for the entomogenous fungi (Araújo et al. 2018; Tasanathai et al. 2019; Tasanathai et al. 2022; Tang et al. 2023a, b). *Ophiocordyceps* Petch is a large genus in the Ophiocordycipitaceae, with approximately 330 accepted species names (Indexfungorum.org. 2023). It was established originally by Petch (Petch 1924, 1931) to accommodate the species of *Cordyceps* Fr. producing asci with conspicuous apical caps and whole ascospores with distinct septation at maturity that do not disarticulate into part-spores. Then *Ophiocordyceps* was used as a subgeneric classification of the genus *Cordyceps* by Kobayasi (1941). *Ophiocordyceps* was restored to the rank of genus to include those *Cordyceps* species in the Ophiocordycipitaceae by Sung et al. (2007). The type of the genus *Ophiocordyceps* was *O.blattae* Petch to be found on cockroach (Blattoidea). *Hirsutella* Pat., *Hymenostilbe* Petch and *Paraisaria* Samson & B.L. Brady are commonly asexual morphs within *Ophiocordyceps*. Species of *Hirsutella* typically produced one to several conidia in a limited number of mucus droplets borne on basally subulate phialides that tapered into slender necks (Gams and Zare 2003). Typically, most of the *Ophiocordyceps* species parasitic to ants and associated with *Hirsutella* included the *O.unilateralis* complex. Entomogenous fungi within *Ophiocordyceps* have a wide range of insect hosts, ranging from solitary beetle larvae to social insects. They are able to colonise insects across 13 orders, including Blattoidea, Coleoptera, Dermaptera, Diptera, Hemiptera, Hymenoptera, Isoptera, Lepidoptera, Mantodea and Megaloptera etc. (Crous et al. 2004; Araújo and Hughes 2016).

Over forty species of *Ophiocordyceps* have been reported from adult ants (Formicidae, Hymenoptera) worldwide (Evans et al. 2011b; Kepler et al. 2011; Luangsa-ard et al. 2011; Kobmoo et al. 2012, 2015; Araújo et al. 2015, 2018; Spatafora et al. 2015; Crous et al. 2016; Wei et al. 2020; Tang et al. 2023a, b). These ant pathogenic fungi with biting behaviour belong to the *O.unilateralis* complex in the *Hirsutella* clade. The *Ophiocordycepsunilateralis* complex species are able to manipulate the ant behaviour by controlling it to leave the nest to die attached on to an ideal location for the fungus to develop, to produce the fruiting body and to begin spore transmission (Hughes et al. 2011). The *Ophiocordycepsunilateralis* complex is widely distributed around the world, for example, Australia, Brazil, China, Colombia, Ghana, Japan, Thailand and USA (Evans and Samson 1982; Evans et al. 2011b; Kepler et al. 2011; Luangsa-ard et al. 2011; Kobmoo et al. 2012, 2015; Araújo et al. 2015, 2018; Spatafora et al. 2015; Crous et al. 2016; Wei et al. 2020; Tang et al. 2023a, b). Although many taxa of the *O.unilateralis* complex have been reported and described in previous studies, there are estimated to be tens or even hundreds of undescribed species worldwide (Evans et al. 2011a). Many cryptic species in the *O.unilateralis* complex need to be further collected globally to explore the diversity of this species complex.

The diagnostic for the *O.unilateralis* complex is the ant biting behaviour, single or (sometimes multiple) stalk(s) arising from the dorsal pronotum of dead ants, with one or multiple lateral cushions from the base to the top along the stroma attached unilaterally (hence the epithet), exhibiting hirsutella-like anamorphs, whole and septate ascospores that do not disarticulate into part-spores that often exhibit secondary germination (capilliconidiophore) (Kobmoo et al. 2012; Araújo et al. 2018; Tang et al. 2023b). In addition, the host species is also a very useful characteristic for identification amongst species. Although the ant identification depends upon morphological features, for the zombie-ants (infected from fungi), some vital characteristics may have been obscured by the pathogenic fungi, therefore posing a challenge to identify the infected ants. With the further application of molecular technology, the mitochondrial cytochrome c oxidase subunit I (*COI*) gene as molecular marker was used for the ant’s phylogenetic studies, for exploring the diversity of the ants and distinguishing species and subspecies in the ant complex (Hebert et al. 2003; Narain et al. 2013; Siddiqui et al. 2019). There are few studies from the zombie-ant fungi elucidating the molecular details or DNA barcodes of these hosts (Tang et al. 2023a, b). In previous studies, the *COI* gene was used to construct a phylogenetic tree of the host ants by Tang et al. (2023b) and for species identification of the hosts. These studies showed that four species of the *O.unilateralis* complex were recorded on the same ant *Camponotus* sp. (Tang et al. 2023b). Evans et al. (2011b) and Araújo et al. (2015, 2018) have suggested that each fungal species seemed to be specifically associated with a given ant species and the host identity used as a proxy for fungal identification. Therefore, reconstructing the host phylogeny is important to understand the evolutionary event between fungi and the ants.

In China, nine species occurring on Formicinae (Formicidae) exhibiting similar behavioural manipulation have been reported in previous studies (Wei et al. 2020; Tang et al. 2023a, b), including *O.acroasca* Hong Yu bis & D.X. Tang, *O.bifertilis* Hong Yu bis & D.X. Tang, *O.subtiliphialida* Hong Yu bis & D.X. Tang, *O.basiasca* Hong Yu bis & D.X. Tang, *O.nuozhaduensis* Hong Yu bis & D.X. Tang, *O.contiispora* Hong Yu bis & D.X. Tang, *O.flabellata* Hong Yu bis & D.X. Tang, *O.lilacina* Hong Yu bis & D.X. Tang and *O.tianshanensis* L. S. Zha, D. P. Wei & K. D. Hyde. Most species were found in subtropical monsoon evergreen broad-leaved forest in southwest China. The two novel species presented herein have been collected from Yunnan Province in China. Based on morphological and phylogenetic characteristics, they were identified as belonging to the core clade of *O.unilateralis*. This study aims to present two novel species of the “zombie-ant fungi” belonging to the *O.unilateralis* core clade, *O.tortuosa* and *O.ansiformis*, from China and to investigate their phylogenetic relationships.

## ﻿Materials and methods

### ﻿Specimen collection

The specimens were collected from south-western China. Collections took place in subtropical monsoon evergreen broad-leaved forest. The ant’s death location from above the ground and the ants attached (biting) to substrate types (e.g. leaf, spine, trunk, moss and base of trunk) were measured and recorded in the field, then all specimens were collected in sterilised plastic containers, transported to the laboratory and examined within the same day if possible or stored at 4 °C. The specimens were deposited in the Yunnan Herbal Herbarium (**YHH**) of Yunnan University.

### ﻿Morphological studies

For ecological characteristics, the quantity of stromata and ascomata per specimen and their colour, size and position were recorded, photographed and examined using a stereomicroscope Olympus SZ61 (Olympus Corporation, Tokyo, Japan). The stromata and the legs from the same ant host were moved for morphological studies. A cryosectioning of the ascoma was performed using a Freezing Microtome HM525NX (Thermo Fisher Scientific, Massachusetts, America). Samples were mounted on a slide with sterile water or lactophenol cotton blue solution for light microscopy examination using an Olympus BX53 (Tokyo, Japan). Micro-morphological characteristics (perithecia, asci, apical caps and ascospores) were examined. The naturally released ascospores and germination events were examined using an Olympus BX53 and the detailed method was based on the research of Araújo et al. (2018).

### ﻿DNA extraction, PCR amplification and sequencing

DNA templates (contains the host and fungus from the same specimen) were obtained directly from fresh specimens using the Plant DNA Isolation Kit (Foregene Co., Ltd., Chengdu, China) according to the manufacturer’s protocols. Polymerase chain reaction (PCR) was used to amplify genetic markers using the following primer pairs: NS1/NS4 for small subunit nuclear ribosomal DNA (SSU) (White et al. 1990), 2218R/983F for translation elongation factor 1-α (*TEF1a*) (Rehner and Buckley 2005), CRPB1/RPB1Cr_oph for partial RNA polymerase II largest subunit gene region (*RPB1*) (Castlebury et al. 2004; Araújo et al. 2018) and LCO1490/HCO2198 for cytochrome oxidase subunit 1 (Hebert et al. 2003).

Each 25 µl-PCR reaction contained 2.5 µl of PCR 10× Buffer (2 mmol/l Mg^2+^) (Transgen Biotech, Beijing, China), 17.25 µl of sterile water, 2 µl of dNTP (2.5 mmol/l), 1 µl of each forward and reverse primer (10 µmol/l), 0.25 µl of Taq DNA polymerase (Transgen Biotech, Beijing, China) and 1 µl of DNA template (500 ng/µl). The PCR reactions were placed in a Bio-Rad T100 thermocycler (Bio-Rad Laboratories Co., Ltd, Shanghai, China) under the following conditions: For SSU, (1) 4 min at 95 °C, (2) 22 cycles of denaturation at 94 °C for 1 min, annealing at 53 °C for 1 min, and extension at 72 °C for 1.3 min, followed by (3) 12 cycles of denaturation at 94 °C for 1 min, annealing at 52 °C for 1 min, and extension at 72 °C for 1.35 min and (4) 8 min at 72 °C (Wang et al. 2015). For *TEF1a*, (1) 4 min at 95 °C, (2) 8 cycles of denaturation at 94 °C for 50 s, annealing at 52 °C for 50 s and extension at 72 °C for 1 min, followed by (3) 30 cycles of denaturation at 94 °C for 50 s, annealing at 51 °C for 50 s and extension at 72 °C for 1 min and (4) 10 min at 72 °C (Wang et al. 2015). For *RPB1*, (1) 4 min at 95 °C, (2) 30 cycles of denaturation at 94 °C for 50 s, annealing at 52 °C for 50 s and extension at 72 °C for 1 min, followed by (3) 8 cycles of denaturation at 94 °C for 50 s, annealing at 51 °C for 50 s and extension at 72 °C for 1 min and (4) 10 min at 72 °C (Wang et al. 2015). For *COI*, (1) 1 min at 95 °C, (2) 5 cycles of denaturation at 94 °C for 1 min, annealing at 50 °C for 1.5 min and extension at 72 °C for 1.5 min, followed by (3) 35 cycles of denaturation at 94 °C for 1 min, annealing at 54 °C for 1.5 min and extension at 72 °C for 1 min and (4) 5 min at 72 °C (Hebert et al. 2003). PCR products were purified using the Gel Band Purifcation Kit (Bio Teke Co., Ltd, Beijing, China) and sequenced by Beijing Genomics Institute (Chongqing, China). All LSU and *RPB2* sequences were downloaded from GenBank.

### ﻿Phylogenetic analyses

#### ﻿Phylogenetic analyses of fungi

To construct a phylogeny of major lineages in *Ophiocordyceps*, most of the DNA sequences used in this work were based on previous phylogenetic studies (Sung et al. 2007; Quandt et al. 2014; Araújo et al. 2018). Phylogenetic analyses were based on sequences of five molecular markers: SSU, LSU, *TEF1a*, *RPB1* and *RPB2*, all of which were downloaded from NCBI (https://www.ncbi.nlm.nih.gov/). Then the nucleotide sequences were combined with those generated in our study (Table 1). Sequences were aligned using ClustalX v.2.0 (Larkin et al. 2007), adjusted manually and then concatenated in BioEdit v.7.1.1 (Hall 1999). ModelFinder (Kalyaanamoorthy et al. 2017) was employed to determine the best fitting likelihood model for Maximum Likelihood (ML) and Bayesian Inference (BI) analyses according to the corrected Akaike Information Criterion (AIC). For ML analyses, tree searches were performed in IQ-tree v.2.1.3 (Nguyen et al. 2015), based on the best-fit model (TIM2+F+I+G4) with 5000 ultrafast bootstraps (Hoang et al. 2017) in a single run. BI analyses were performed in MrBayes v.3.2.7 (Ronquist et al. 2012). The BI search was based on the GTR+F+I+G4 model. Four Markov Chain Monte Carlo chains (one cold, three heated) were run, each beginning with a random tree and sampling one tree every 100 generations of 2,000,000 generations and the first 25% of samples were discarded as burn-in. The tree was visualised with its Maximum-Likelihood bootstrap proportions (ML-BS) and Bayesian posterior probability (BI-BPP) in Figtree v.1.4.3. Adobe Illustrator CS6 was used for editing.

**Table 1.** The taxa, GenBank accession numbers and host information in this study.

Species name	Voucher information	Host	SSU	LSU	*TEF1α*	*RPB1*	*RPB2*	Reference
*Hirsutella* sp.	NHJ 12525	Hemiptera	EF469125	EF469078	EF469063	EF469092	EF469111	Sung et al. (2007)
	OSC 128575	Hemiptera	EF469126	EF469079	EF469064	EF469093	EF469110	Sung et al. (2007)
*Ophiocordycepsacicularis*	ARSEF 5692	Coleoptera	DQ522540	DQ518754	DQ522322	DQ522368	DQ522418	Spatafora et al. (2007)
	OSC 128580	Coleoptera	DQ522543	DQ518757	DQ522326	DQ522371	DQ522423	Spatafora et al. (2007)
*Ophiocordycepsacroasca*	YFCC 9049	*Camponotus* sp.	ON555837	ON555918	ON567757	ON568677	ON568130	Tang et al. (2023b)
	YFCC 9019	*Camponotus* sp.	ON555838	ON555919	ON567758	ON568678	ON568131	Tang et al. (2023b)
	YFCC 9017	*Camponotus* sp.	ON555839	ON555920	ON567759	ON568679	ON568132	Tang et al. (2023b)
	YFCC 9018	*Camponotus* sp.	ON555840	ON555921	ON567760	ON568680	ON568133	Tang et al. (2023b)
	YFCC 9016	*Camponotus* sp.	ON555841	ON555922	ON567761	ON568681	ON568134	Tang et al. (2023b)
	YHH 20122	*Camponotus* sp.	ON555842		ON567762	ON568682		Tang et al. (2023b)
*Ophiocordycepsalbacongiuae*	RC20	*Camponotus* sp.	KX713633		KX713670			Araújo et al. (2018)
*Ophiocordycepsannullata*	CEM 303	Coleoptera	KJ878915	KJ878881	KJ878962	KJ878995		Quandt et al. (2014)
*Ophiocordycepsaphodii*	ARSEF 5498	Coleoptera	DQ522541	DQ518755	DQ522323		DQ522419	Spatafora et al. (2007)
*Ophiocordycepsaustralis*	HUA 186097	*Pachycondyla* sp.	KC610786	KC610765	KC610735	KF658662		Sanjuan et al. (2015)
*Ophiocordycepsbasiasca*	YHH 20191	*Camponotus* sp.	ON555828	ON555910	ON567748	ON568672	ON568121	Tang et al. (2023b)
*Ophiocordycepsbifertilis*	YFCC 9012	*Polyrhachis* sp.	ON555843	ON555923	ON567763	ON568143	ON568135	Tang et al. (2023b)
	YHH 20162	*Polyrhachis* sp.	ON555844		ON567764	ON568144		Tang et al. (2023b)
	YHH 20163	*Polyrhachis* sp.	ON555845	ON555924	ON567765	ON568145	ON568136	Tang et al. (2023b)
	YHH 20164	*Polyrhachis* sp.	ON555846		ON567766	ON568146		Tang et al. (2023b)
	YFCC 9048	*Polyrhachis* sp.	ON555847	ON555925	ON567767	ON568147	ON568137	Tang et al. (2023b)
	YFCC 9013	*Polyrhachis* sp.	ON555848	ON555926	ON567768	ON568148	ON568138	Tang et al. (2023b)
*Ophiocordycepsblakebarnesii*	MISSOU5	*Camponotus* sp.	KX713641	KX713610	KX713688	KX713716		Araújo et al. (2018)
	MISSOU4	*Camponotus* sp.	KX713642	KX713609	KX713685	KX713715		Araújo et al. (2018)
*Ophiocordycepsbrunneipunctata*	OSC 128576	Coleoptera	DQ522542	DQ518756	DQ522324	DQ522369	DQ522420	Spatafora et al. (2007)
*Ophiocordycepsbuquetii*	HMAS_199617	Hymenoptera	KJ878940	KJ878905	KJ878985	KJ879020		Quandt et al. (2014)
*Ophiocordycepscamponoti-balzani*	G143	*Camponotusbalzani*	KX713658	KX713595	KX713690	KX713705		Araújo et al. (2018)
	G104	*Camponotusbalzani*	KX713660	KX713593	KX713689	KX713703		Araújo et al. (2018)
*Ophiocordycepscamponoti-bispinosi*	OBIS5	*Camponotusbispinosus*	KX713636	KX713616	KX713693	KX713721		Araújo et al. (2018)
	OBIS4	*Camponotusbispinosus*	KX713637	KX713615	KX713692	KX713720		Araújo et al. (2018)
*Ophiocordycepscamponoti-chartificis*	MF080	*Camponotuschartifex*	MK874744		MK863824			Araújo et al. (2018)
*Ophiocordycepscamponoti-femorati*	FEMO2	*Camponotusfemoratus*	KX713663	KX713590	KX713678	KX713702		Araújo et al. (2018)
*Ophiocordycepscamponoti-floridani*	Flo4	*Camponotusfemoratus*	KX713662	KX713591				Araújo et al. (2018)
	Flx2	*Camponotusfemoratus*		KX713592	KX713674			Araújo et al. (2018)
*Ophiocordycepscamponoti-hippocrepidis*	HIPPOC	*Camponotushippocrepis*	KX713655	KX713597	KX713673	KX713707		Araújo et al. (2018)
*Ophiocordycepscamponoti-indiani*	INDI2	*Camponotusindianus*	KX713654	KX713598				Araújo et al. (2018)
*Ophiocordycepscamponoti-leonardi*	C27	*Camponotusleonardi*			JN819019			Kobmoo et al. (2012)
	C25	*Camponotusleonardi*			JN819029			Kobmoo et al. (2012)
*Ophiocordycepscamponoti-nidulantis*	NIDUL2	*Camponotusnidulans*	KX713640	KX713611	KX713669	KX713717		Araújo et al. (2018)
*Ophiocordycepscamponoti-novogranadensis*	Mal63	*Camponotusnovogranadensis*	KX713648	KX713603				Araújo et al. (2018)
	Mal4	*Camponotusnovogranadensis*	KX713649	KX713602				Araújo et al. (2018)
*Ophiocordycepscamponoti-renggeri*	RENG2	*Camponotusrenggeri*	KX713632		KX713672			Araújo et al. (2018)
	ORENG	*Camponotusrenggeri*	KX713634	KX713617	KX713671			Araújo et al. (2018)
*Ophiocordycepscamponoti-rufipedis*	G177	*Camponotusrufipes*	KX713657	KX713596	KX713680			Araújo et al. (2018)
	G108	*Camponotusrufipes*	KX713659	KX713594	KX713679	KX713704		Araújo et al. (2018)
*Ophiocordycepscamponoti-saundersi*	C40	*Camponotussaundersi*	KJ201519		JN819012			Kobmoo et al. (2012)
*Ophiocordycepscamponoti-saundersi*	Co19	*Camponotussaundersi*			JN819018			Kobmoo et al. (2012)
*Ophiocordycepscitrina*	TNS F18537	Hemiptera		KJ878903	KJ878983		KJ878954	Quandt et al. (2014)
*Ophiocordycepsclavata*	CEM 1762	Coleoptera	KJ878916	KJ878882	KJ878963	KJ878996		Quandt et al. (2014)
*Ophiocordycepscochlidiicola*	HMAS_199612	Lepidoptera	KJ878917	KJ878884	KJ878965	KJ878998		Quandt et al. (2014)
*Ophiocordycepscontiispora*	YFCC 9025	*Camponotus* sp.	ON555829	ON555911	ON567749	ON568139	ON568122	Tang et al. (2023b)
	YHH 20145	*Camponotus* sp.	ON555830		ON567750	ON568140	ON568123	Tang et al. (2023b)
	YFCC 9026	*Camponotus* sp.	ON555831	ON555912	ON567751	ON568141	ON568124	Tang et al. (2023b)
	YFCC 9027	*Camponotus* sp.	ON555832	ON555913	ON567752	ON568142	ON568125	Tang et al. (2023b)
*Ophiocordycepscurculionum*	OSC 151910	Coleoptera	KJ878918	KJ878885		KJ878999		Quandt et al. (2014)
*Ophiocordycepsdaceti*	MF01	*Dacetonarmigerum*		KX713604	KX713667			Araújo et al. (2018)
*Ophiocordycepsdipterigena*	OSC 151911	Diptera	KJ878919	KJ878886	KJ878966	KJ879000		Quandt et al. (2014)
	OSC 151912	Diptera	KJ878920	KJ878887	KJ878967	KJ879001		Quandt et al. (2014)
*Ophiocordycepsflabellata*	YFCC 8795	Hymenoptera (*Camponotus* sp.)	OL310721	OL310724	OL322688	OL322687	OL322695	Tang et al. (2023a)
	YFCC 8796	Hymenoptera (*Camponotus* sp.)	OL310722	OL310723	OL322692	OL322689	OL322696	Tang et al. (2023a)
	YHH 20038	Hymenoptera (*Camponotus* sp.)			OL322694	OL322691		Tang et al. (2023a)
	YHH 20037	*Camponotus* sp.			OL322693	OL322690	OL322697	Tang et al. (2023a)
*Ophiocordycepsformosana*	TNM F13893	Coleoptera	KJ878908		KJ878956	KJ878988	KJ878943	Quandt et al. (2014)
*Ophiocordycepsformicarum*	TNS F18565	Hymenoptera	KJ878921	KJ878888	KJ878968	KJ879002	KJ878946	Quandt et al. (2014)
*Ophiocordycepsforquignonii*	OSC 151902	Diptera	KJ878912	KJ878876		KJ878991	KJ878945	Quandt et al. (2014)
	OSC 151908	Diptera	KJ878922	KJ878889		KJ879003	KJ878947	Quandt et al. (2014)
*Ophiocordyceps* sp.	Gh41	*Polyrhachis* sp.	KX713656		KX713668	KX713706		Araújo et al. (2018)
*Ophiocordycepshalabalaensis*	MY1308	*Camponotusgigus*	KM655825		GU797109			Kobmoo et al. (2015)
*Ophiocordycepshalabalaensis*	MY5151	*Camponotusgigas*	KM655826		GU797110			Kobmoo et al. (2015)
*Ophiocordycepsansiformis*	YHH 2210007	*Colobopsis* sp.	OR345230		OR098435	OR351952		This study
*Ophiocordycepsirangiensis*	OSC 128577	Hymenoptera	DQ522546	DQ518760	DQ522329	DQ522374	DQ522427	Spatafora et al. (2007)
	OSC 128578	Hymenoptera	DQ522556	DQ518770	DQ522345	DQ522391	DQ522445	Spatafora et al. (2007)
	OSC 128579	Hymenoptera	EF469123	EF469076	EF469060	EF469089	EF469107	Sung et al. (2007)
*Ophiocordycepskimflemingiae*	SC30	*Camponotuscastaneus*/*americanus*	KX713629	KX713622	KX713699	KX713727		Araújo et al. (2018)
	SC09B	*Camponotuscastaneus*/*americanus*	KX713631	KX713620	KX713698	KX713724		Araújo et al. (2018)
*Ophiocordycepskniphofioides*	HUA 186148	Hymenoptera	KC610790	KF658679	KC610739	KF658667	KC610717	Sanjuan et al. (2015)
*Ophiocordycepskonnoana*	EFCC 7295	Coleoptera	EF468958			EF468862	EF468915	Sung et al. (2007)
	EFCC 7315	Coleoptera	EF468959		EF468753	EF468861	EF468916	Sung et al. (2007)
*Ophiocordycepslilacina*	YHH 2210001	*Polyrhachis* sp.	OP782343		OP796856	OP796861		Tang et al. (2023a)
	YHH 2210002	*Polyrhachis* sp.	OP782344		OP796857	OP796862		Tang et al. (2023a)
*Ophiocordycepslloydii*	OSC 151913	Hymenoptera	KJ878924	KJ878891	KJ878970	KJ879004	KJ878948	Quandt et al. (2014)
*Ophiocordycepslongissima*	TNS F18448	Hemiptera	KJ878925	KJ878892	KJ878971	KJ879005		Quandt et al. (2014)
	HMAS_199600	Hemiptera	KJ878926		KJ878972	KJ879006	KJ878949	Quandt et al. (2014)
*Ophiocordycepsmelolonthae*	OSC 110993	Coleoptera	DQ522548	DQ518762	DQ522331	DQ522376		Spatafora et al. (2007)
	Ophgrc679	Coleoptera		KC610768	KC610744	KF658666		Sanjuan et al. (2015)
*Ophiocordycepsmonacidis*	MF74C	*Dolichoderusbispinosus*	KX713646	KX713606				Araújo et al. (2018)
	MF74	*Dolichoderusbispinosus*	KX713647	KX713605		KX713712		Araújo et al. (2018)
*Ophiocordycepsmyrmecophila*	CEM1710	Hymenoptera	KJ878928	KJ878894	KJ878974	KJ879008		Quandt et al. (2014)
*Ophiocordycepsnaomipierceae*	DAWKSANT	Polyrhachiscf.robsonii	KX713664	KX713589		KX713701		Araújo et al. (2018)
*Ophiocordycepsneovolkiana*	OSC 151903	Coleoptera	KJ878930	KJ878896	KJ878976	KJ879010		Quandt et al. (2014)
*Ophiocordycepsnigrella*	EFCC 9247		EF468963	EF468818	EF468758	EF468866	EF468920	Sung et al. (2007)
*Ophiocordycepsnooreniae*	BRIP 55363	Chariomyrmacf.hookeri and *Polyrhachislydiae*	NG065096	NG059720	KX673812		KX673809	Crous et al. (2016)
	BRIP 64868	Polyrhachiscf.hookeri and *Polyrhachislydiae*	KX961142		KX961143			Crous et al. (2016)
*Ophiocordycepsnutans*	OSC 110994	Hemiptera	DQ522549	DQ518763	DQ522333	DQ522378		Spatafora et al. (2007)
*Ophiocordycepsnuozhaduensis*	YHH 20168	*Camponotus* sp.	ON555849	ON555927	ON567769	ON568683		Tang et al. (2023b)
	YHH 20169	*Camponotus* sp.	ON555850	ON555928	ON567770	ON568684		Tang et al. (2023b)
*Ophiocordycepsodonatae*	TNS F18563	Odonata		KJ878877		KJ878992		Quandt et al. (2014)
	TNS F27117	Odonata		KJ878878				Quandt et al. (2014)
*Ophiocordycepsoecophyllae*	OECO1	*Oecophyllassmaragdina*	KX713635					Araújo et al. (2018)
*Ophiocordycepsootakii*	J14	*Polyrhachismoesta*	KX713651		KX713682	KX713709		Araújo et al. (2018)
*Ophiocordycepsootakii*	J13	*Polyrhachismoesta*	KX713652	KX713600	KX713681	KX713708		Araújo et al. (2018)
*Ophiocordycepsponerinarum*	HUA 186140	*Paraponeraclavata*	KC610789	KC610767	KC610740	KF658668		Araújo et al. (2018)
*Ophiocordycepspolyrhachis-furcata*	P39	*Polyrhachisfurcata*	KJ201504		JN819003			Kobmoo et al. (2012)
	P51	*Polyrhachisfurcata*	KJ201505		JN819000			Kobmoo et al. (2012)
*Ophiocordycepspulvinata*	TNS-F-30044	*Camponotusobscuripes*	GU904208		GU904209	GU904210		Kepler et al. (2011)
*Ophiocordycepspurpureostromata*	TNS F18430	Coleoptera	KJ878931	KJ878897	KJ878977	KJ879011		Araújo et al. (2018)
*Ophiocordycepsrami*	MY6736	*Camponotus* sp.	KM655823		KJ201532			Kobmoo et al. (2015)
	MY6738	*Camponotus* sp.	KM655824		KJ201534			Kobmoo et al. (2015)
*Ophiocordycepsravenelii*	OSC 151914	Coleoptera	KJ878932		KJ878978	KJ879012	KJ878950	Quandt et al. (2014)
*Ophiocordycepsrhizoidea*	NHJ 12529	Coleoptera	EF468969	EF468824	EF468765	EF468872	EF468922	Sung et al. (2007)
	NHJ 12522	Coleoptera	EF468970	EF468825	EF468764	EF468873	EF468923	Sung et al. (2007)
*Ophiocordycepssatoi*	J19	*Polyrhachislamellidens*	KX713650	KX713601	KX713684	KX713710		Araújo et al. (2018)
	J7	*Polyrhachislamellidens*	KX713653	KX713599	KX713683	KX713711		Araújo et al. (2018)
	YFCC 8807	*Polyrhachis* sp.	OP782340	OP782345	OP796853	OP796858	OP796863	Tang et al. (2023a)
	YFCC 8809	*Polyrhachis* sp.	OP782341	OP782346	OP796854	OP796859	OP796864	Tang et al. (2023a)
	YFCC 8810	*Polyrhachis* sp.	OP782342	OP782347	OP796855	OP796860	OP796865	Tang et al. (2023a)
*Ophiocordycepssepta*	Pur1	*Camponotus* sp.			KJ201528			Araújo et al. (2018)
	Pur2	*Camponotus* sp.			KJ201529			Araújo et al. (2018)
	C41	*Camponotus* sp.			JN819037			Kobmoo et al. (2015)
*Ophiocordycepssinensis*	EFCC 7287	Lepidoptera	EF468971	EF468827	EF468767	EF468874	EF468924	Sung et al. (2007)
*Ophiocordycepssobolifera*	KEW 78842	Hemiptera	EF468972	EF468828		EF468875	EF468925	Sung et al. (2007)
*Ophiocordycepssphecocephala*	OSC 110998	Hymenoptera	DQ522551	DQ518765	DQ522336	DQ522381	DQ522432	Spatafora et al. (2007)
*Ophiocordycepsstylophora*	OSC 111000	Coleoptera	DQ522552	DQ518766	DQ522337	DQ522382	DQ522433	Spatafora et al. (2007)
	OSC 110999	Coleoptera	EF468982	EF468837	EF468777	EF468882	EF468931	Sung et al. (2007)
*Ophiocordycepssubtiliphialida*	YFCC 8815	*Camponotus* sp.	ON555833	ON555914	ON567753	ON568673	ON568126	Tang et al. (2023b)
	YFCC 8814	*Camponotus* sp.	ON555834	ON555915	ON567754	ON568674	ON568127	Tang et al. (2023b)
	YFCC 8816	*Camponotus* sp.	ON555835	ON555916	ON567755	ON568675	ON568128	Tang et al. (2023b)
	YFCC 8817	*Camponotus* sp.	ON555836	ON555917	ON567756	ON568676	ON568129	Tang et al. (2023b)
*Ophiocordycepstianshanensis*	MFLU 19-1207	*Camponotusjaponicus*	MN025409	MN025407	MK992784			Wei et al. (2020)
	MFLU 19-1208	*Camponotusjaponicus*	MN025410	MN025408	MK992785			Wei et al. (2020)
*Ophiocordycepstortuosa*	YHH 2210003	*Colobopsis* sp.			OR098431	OR098436		This study
	YHH 2210004	*Colobopsis* sp.	OR067858		OR098432	OR098437		This study
	YHH 2210005	*Colobopsis* sp.	OR067859		OR098433	OR098438		This study
	YHH 2210006	*Colobopsis* sp.			OR098434	OR098439		This study
*Ophiocordycepstricentri*	CEM 160	Hemiptera	AB027330	AB027376				Nikoh and Fukatsu (2000)
*Ophiocordycepsunilateralis*	VIC 44303	*Camponotussericeiventris*	KX713628	KX713626	KX713675	KX713730		Araújo et al. (2018)
	VIC 44354	*Camponotussericeiventris*	KX713627		KX713676	KX713731		Araújo et al. (2018)
*Ophiocordycepsyakusimensis*	HMAS_199604	Hemiptera	KJ878938	KJ878902		KJ879018	KJ878953	Quandt et al. (2014)
*Paraisariaamazonica*	HUA 186113	Orthoptera	KJ917566			KP212903	KM411980	Sanjuan et al. (2015)
*Paraisariagracilis*	EFCC 8572	Lepidoptera	EF468956	EF468811	EF468751	EF468859	EF468912	Sung et al. (2007)
	EFCC 3101	Lepidoptera	EF468955	EF468810	EF468750	EF468858	EF468913	Sung et al. (2007)
*Paraisariaheteropoda*	OSC 106404	Hemiptera	AY489690	AY489722	AY489617	AY489651		Castlebury et al. (2004)
*Tolypocladiuminflatum*	OSC 71235	Coleoptera	EF469124	EF469077	EF469061	EF469090	EF469108	Sung et al. (2007)
*Tolypocladiumophioglossoides*	CBS 100239	*Elaphomyces* sp.	KJ878910	KJ878874	KJ878958	KJ878990	KJ878944	Quandt et al. (2014)

#### ﻿Phylogenetic analyses of host

Phylogenetic analyses were based on *COI* gene sequences. Most of the DNA sequences used in this work were based on previous phylogenetic studies (Mezger and Moreau 2015; Tang et al. 2023a, 2023b) and partial sequences were retrieved using the BLASTn searches in GenBank. The nucleotide sequences downloaded from NCBI were then combined with those generated in our study. Information on specimens and GenBank accession numbers are listed in Table 2. Sequences were initially aligned using ClustalX, manually adjusted and then concatenated in BioEdit. ModelFinder was used to select the best-fitting likelihood model (GTR+F+I+G4) for ML analyses and BI analyses according to the AIC. The host dataset used the same tree search setting as for the fungi phylogenetic inference.

**Table 2.** Specimen and GenBank accession numbers information for *COI* genes used in this study.

Species name	Voucher information	GenBank number	Reference
*Camponotusamericanus*	YNH-005	MZ331828	Unpublished
*Camponotuscastaneus*	BIOUG03675-H07	KJ208900	Unpublished
	BIOUG03675-H04	KJ445248	Unpublished
*Camponotusclaripes*	AECT	JN134855	Unpublished
*Camponotusrenggeri*	Creng_1_B	KP101600	Unpublished
*Colobopsisrufipes*	BIOUG24424-D11	OM314604	Unpublished
*Camponotussimulans*	AFR-CND-2010-47-F02	JN270684	Unpublished
*Camponotus* sp.	CASENT0441197-D01	GU710187	Unpublished
	CASENT0043700-D01	KF200199	Unpublished
	CAMPO014	MH290634	Unpublished
	CASENT0000633-D01	HM373060	Unpublished
	YHH20648	OP783989	Tang et al. (2023a)
	YHH 20605	OP353540	Tang et al. (2023b)
	YHH 20606	OP353541	Tang et al. (2023b)
	YHH 20607	OP353542	Tang et al. (2023b)
	YHH 20608	OP353543	Tang et al. (2023b)
	YHH 20609	OP353544	Tang et al. (2023b)
	YHH 20610	OP353545	Tang et al. (2023b)
	YHH 20611	OP353546	Tang et al. (2023b)
	YHH 20612	OP353547	Tang et al. (2023b)
	YHH 20168	OP353548	Tang et al. (2023b)
	YHH 20191	OP353549	Tang et al. (2023b)
	YHH 20122	OP353539	Tang et al. (2023b)
*Colobopsis* sp.	YHH 2210006	OR068149	This study
	YHH 2210007	OR068150	This study
*Camponotusspanis*	G191388	OM420293	Unpublished
*Camponotussericeiventris*	BIOUG13980-G06	OM558348	Unpublished
	BIOUG24738-E05	OM556713	Unpublished
*Camponotussexguttatus*	CASENT0612243	JF863527	Unpublished
*Colobopsisbadia*	TUCIM:6601	MF993268	Laciny et al. (2018)
*Colobopsisexplodens*	TUCIM:5080	MF993254	Unpublished
*Colobopsissaundersi*		BK012313	Allio et al. (2020)
*Colobopsisvitreus*	gvc13410-1L	HM914891	Unpublished
	gvc13412-1L	HM914893	Unpublished
*Camponotuswiederkehri*	AEKB	JN134865	Unpublished
*Dacetonarmigerum*	USNM:ENT:01566820	MW983875	Unpublished
*Oecophyllasmaragdina*	CSM0633	KM348201	Mezger and Moreau (2015)
*Polyrhachisabbreviata*	CSM0776	KM348230	Mezger and Moreau (2015)
*Polyrhachisanderseni*	ANA42	KM348248	Mezger and Moreau (2015)
*Polyrhachisandromache*	FMNH-INS_2842051	KM348264	Mezger and Moreau (2015)
*Polyrhachisammon*	RA0751	KY939110	Unpublished
*Polyrhachisaurea*	RA0750	KM348211	Mezger and Moreau (2015)
*Polyrhachisaustralis*	RA0757	KM348231	Unpublished
*Polyrhachisarnoldiisolate*	NDA40	MK591916	Unpublished
*Polyrhachisbeccari*	FMNH-INS_2842133	KM348266	Mezger and Moreau (2015)
*Polyrhachisbeccari*	FMNH-INS_2842169	KM348265	Mezger and Moreau (2015)
*Polyrhachisbrevinoda*	CSM2831	KY939023	Mezger and Moreau (2015)
	CSM0773	KM348232	Mezger and Moreau (2015)
*Polyrhachiscarbonaria*	FMNH-INS_2842101	KM348267	Mezger and Moreau (2015)
Polyrhachiscf.bismarckensis	FMNH-INS 2842022	KM348331	Mezger and Moreau (2015)
*Polyrhachiscupreata*	CSM1015	KY939064	Unpublished
	CSM0682	KY939056	Unpublished
*Polyrhachiscyphonota*	FMNH-INS_2842221	KM348234	Mezger and Moreau (2015)
*Polyrhachisdanum*	CSM1841	KM348235	Mezger and Moreau (2015)
*Polyrhachisdelecta*	CSM0965	KY939013	Unpublished
*Polyrhachisflavibasis*	RA0766	KM348203	Mezger and Moreau (2015)
	RA0763	KY939081	Unpublished
*Polyrhachisfurcata*	YB-KHC51412	MN618329	Unpublished
*Polyrhachisgagates*	FMNH-INS_2842213	KM348270	Mezger and Moreau (2015)
*Polyrhachishexacantha*	FMNH-INS_2842006	KM348204	Mezger and Moreau (2015)
*Polyrhachishookeri*	RA0747	KM348215	Mezger and Moreau (2015)
*Polyrhachisillaudata*	FMNH-INS_2842112	KM348275	Mezger and Moreau (2015)
	FMNH-INS_2842222	KM348271	Mezger and Moreau (2015)
	GXJX0141	JQ681065	Unpublished
*Polyrhachisjianghuaensis*	GXBL0006	JQ681069	Unpublished
*Polyrhachislatharis*	FMNH-INS_2842062	KM348278	Mezger and Moreau (2015)
*Polyrhachislamellidens*	NSMK-IN-170100347	OL663445	Unpublished
*Polyrhachislepida*	CSM1877	KM348241	Mezger and Moreau (2015)
	CSM1807	KM348239	Mezger and Moreau (2015)
*Polyrhachislucidula*	G160084	OM420302	Unpublished
*Polyrhachismackayi*	CSM0804	KM348242	Mezger and Moreau (2015)
*Polyrhachismonteithi*	CSM0754	KY939009	Unpublished
*Polyrhachismucronata*	RA1154	KM348338	Mezger and Moreau (2015)
	RA1158	KM348339	Mezger and Moreau (2015)
	RA1164	KM348340	Mezger and Moreau (2015)
	CSM0696a	KM348337	Mezger and Moreau (2015)
*Polyrhachisnigropilosa*	FMNH-INS_2842045	KM348284	Mezger and Moreau (2015)
*Polyrhachisnoesaensis*	FMNH-INS_2842106	KM348285	Mezger and Moreau (2015)
*Polyrhachisobesior*	FMNH-INS_2842054	KM348286	Mezger and Moreau (2015)
*Polyrhachisornata*	CSM0797	KM348255	Mezger and Moreau (2015)
	CSM0842	KY939061	Unpublished
*Polyrhachisproxima*	FMNH-INS_2842042	KM348289	Mezger and Moreau (2015)
	FMNH-INS_2842129	KM348288	Mezger and Moreau (2015)
*Polyrhachisrastellata*	FMNH-INS_2841999	KM348244	Mezger and Moreau (2015)
*Polyrhachisrobsoni*	CSM1050	KY939017	Unpublished
*Polyrhachissaevissima*	FMNH-INS_2842115	KM348345	Mezger and Moreau (2015)
*Polyrhachisschistacea*	FMNH-INS_2842058	KM348297	Mezger and Moreau (2015)
	FMNH-INS_2842071	KM348294	Mezger and Moreau (2015)
	FMNH-INS_2842067	KM348293	Mezger and Moreau (2015)
*Polyrhachisschoopae*	CSM0626b	KM348218	Mezger and Moreau (2015)
*Polyrhachis* sp.	FMNH-INS_2842139	KM348305	Mezger and Moreau (2015)
	FMNH-INS_2842198	KM348309	Mezger and Moreau (2015)
	FMNH-INS_2842195	KM348308	Mezger and Moreau (2015)
	FMNH-INS_2842179	KM348300	Mezger and Moreau (2015)
	FMNH-INS_2842190	KM348304	Mezger and Moreau (2015)
	FMNH-INS_2842193	KM348310	Mezger and Moreau (2015)
	FMNH-INS_2842194	KM348307	Mezger and Moreau (2015)
	FMNH-INS_2842074	KM348226	Mezger and Moreau (2015)
	FMNH-INS_2842082	KM348306	Mezger and Moreau (2015)
	FMNH-INS_2842039	KM348311	Mezger and Moreau (2015)
*Polyrhachis* sp.	CSM2738	KM348302	Mezger and Moreau (2015)
	FMNH-INS_2842043	KM348246	Mezger and Moreau (2015)
	RA0779	KY939027	Unpublished
	FMNH-INS_2842044	KM348350	Mezger and Moreau (2015)
	FMNH-INS_2842078	KM348314	Mezger and Moreau (2015)
	FMNH-INS_2842032	KM348313	Mezger and Moreau (2015)
	FMNH-INS_2842103	KM348315	Mezger and Moreau (2015)
	YHH 20635	OP783990	Tang et al. (2023a)
	YHH 20636	OP783991	Tang et al. (2023a)
	YHH 20637	OP783992	Tang et al. (2023a)
	YHH 20638	OP783993	Tang et al. (2023a)
	YHH 20639	OP783994	Tang et al. (2023a)
	YHH 20640	OP783995	Tang et al. (2023a)
	YHH 20641	OP783996	Tang et al. (2023a)
	YHH 20642	OP783997	Tang et al. (2023a)
	YHH 20643	OP783998	Tang et al. (2023a)
	YHH 20644	OP783999	Tang et al. (2023a)
	YHH 20645	OP784000	Tang et al. (2023a)
	YHH 20646	OP784001	Tang et al. (2023a)
	YHH 20647	OP784002	Tang et al. (2023a)
	YHH 20162	OP353532	Tang et al. (2023b)
	YHH 20163	OP353533	Tang et al. (2023b)
	YHH 20164	OP353534	Tang et al. (2023b)
	YHH 20601	OP353535	Tang et al. (2023b)
	YHH 20602	OP353536	Tang et al. (2023b)
	YHH 20603	OP353537	Tang et al. (2023b)
	YHH 20604	OP353538	Tang et al. (2023b)
*Polyrhachistubifera*	CSM1108	KY939104	Unpublished
*Polyrhachisturneri*	CSM0827	KM348260	Mezger and Moreau (2015)
*Polyrhachisvillipes*	FMNH-INS_28421186	KM348316	Mezger and Moreau (2015)
*Polyrhachisviscosa*	FMNH-INS_2842064	KM348317	Mezger and Moreau (2015)

## ﻿Results

### ﻿Phylogenetic analysis of fungi

Combining single gene trees (SSU, *TEF1a*, *RPB1*) in a concatenated tree, using morphological features for comparison, enabled identification of two new species (*O.tropiosa* and *O.ansiformis*). We have inferred the phylogeny, based on each single gene (SSU, *TEF1a*, *RPB1*) and present the details below. *Ophiocordycepstortuosa* was recovered as sister to *O.lilacina* (BS = 88%) (Suppl. material 1) and the relationship between *O.tortuosa* and *O.contiispora* was recovered with strong support (BS = 100%), based on *TEF1a* or *RPB1* (Suppl. materials 2, 3). Sequences of *Ophiocordycepsansiformis*, *O.subtiliphialida*, *O.contiispora* and *O.basiasca* were clustered together into a clade with weak bootstrap support (BS = 49%), based on SSU (Suppl. material 1), *O.ansiformis* was recovered sister to *O.tortuosa* + *O.contiispora* with weak to strong support (BS = 63–91%), based on *TEF1a* or *RPB1* (Suppl. materials 2, 3).

For the concatenated tree (SSU, LSU, *TEF1a*, *RPB1* and *RPB2*), the alignment comprised 143 taxa (Table 1). *Tolypocladiumophioglossoides* CBS 100239 and *T.inflatum* OSC 71235 were used as the outgroup taxa. The final trimmed five genetic marker matrix contained 4,827 bp, including 1,059 bp for SSU, 966 bp for LSU, 967 bp for *TEF1a*, 762 bp for *RPB1* and 1,073 bp for *RPB2*. This matrix has 2,688 distinct patterns, 1,699 parsimony-informative, 410 singleton sites and 2,718 constant sites. The likelihood of the best scoring IQ tree was −54,690.799. The best-fit model TIM2+F+I+G4 was used for Maximum Likelihood analysis and the GTR+F+I+G4 model was used for the Bayesian analysis. The generic level relationships of ML and BI trees were topologically similar. In agreement with the previous study by Araújo et al. (2018), phylogenetic analyses showed that the *Hirsutella* ant pathogen consisted of three major groups, i.e. *O.unilateralis* core clade, *O.oecophyllae* and *O.kniphofioides* sub-clade. The *O.unilateralis* core clade included 38 species and was strongly supported (BS = 100%, BPP = 100%), *O.oecophyllae* branched as its sister taxon with BS = 98%, BPP = 80%. The subclade *O.kniphofioides* was sister to the core clade *O.unilateralis* + *O.oecophyllae* clade with strong support (BS = 100%, BPP = 98%). The phylogenetic analysis indicated that the two species in this study were clustered together in the *O.unilateralis* core clade within the Southeast Asian clade and that the two new taxa formed distinct lineages from the other species, respectively. The sister relationships between *O.tortuosa* and *O.contiispora* were recovered with strong support (BS = 100%, BPP = 100%) and obtained the same topological structure as the single gene (*TEF1a* and *RPB1*) tree (Suppl. materials 2 and 3). *Ophiocordycepsansiformis* was recovered sister to *O.tortuosa* + *O.contiispora* with strong support (BS = 85%, BPP = 95%) and also obtained the same topological structure as the single gene (*TEF1a* and *RPB1*) tree (Suppl. materials 2, 3).

### ﻿Phylogenetic analysis of host ants

The alignment consisted of 131 taxa (Table 2). *Dacetonarmigerum* USNM was used as the outgroup taxa. The final trimmed *COI* genetic marker matrix contained 660 bp. The matrix had 389 distinct patterns, 309 parsimony-informative, 42 singleton sites and 309 constant sites. The likelihood of the best scoring IQ tree was −16,415.047. The best-fit model GTR+F+I+G4 was used for Maximum Likelihood analysis and Bayesian analysis. The generic level relationships of ML and BI trees were topologically similar.

Phylogenetic analyses showed that the genera *Colonopsis* (BS = 98%, BPP = 100%) and *Polyrhachis* (BS = 91%, BPP = 99%) within Formicinae formed each a monophyletic clade with strong supports and statistical topology. The phylogenetic analysis indicated that the hosts *Colonopsis* sp. (YHH 2210006 and YHH 2210007) formed a clade and were infected by both *O.tortuosa* and *O.ansiformis* (Fig. 2). Ants infected with the two new fungi were identified by morphological features and *COI* phylogenetic analysis as belonging to the same host, *Colonopsis* sp. The ant species could not be further identified because the ant characteristics were not obvious. Interestingly, these ant pathogenic fungi, including *O.basiasca*, *O.contiispora*, *O.acroasca* and *O.subtiliphialida* also parasitised on the same host (*Camponotus* sp. YHH 20606, 20609, 20608, 20611, 20607, 20191, 20610, 20122, 20605 and 20612) in Tang et al. studies (2023b). The results in this work suggest that the fungal pathogen infects the same host as *Colonopsis* sp. and that the two species may share the same niche.

### ﻿Taxonomy

#### ﻿ Ophiocordyceps tortuosa

Taxon classificationFungiHypocrealesOphiocordycipitaceae

Hong Yu bis, D.X. Tang & J. Zhao sp. nov.

6C6D9418-4ECB-5993-B59B-AA3E2E670754

849060

Fig. 3

##### Etymology.

Tortuosa = tortuous, the epithet referred to the “tortuous” arrangement of ascospores in the asci.

##### Diagnosis.

The difference between *Ophiocordycepstortuosa* and related species is that *Ophiocordycepstortuosa* produces lanceolate and obvious separate ascospores, while *O.contiispora* produces fusiform and no obvious separate ascospores.

##### Holotype.

China, Yunnan Province, Puer City, Simao District. Infected *Colobopsis* sp. (Formicinae) biting into a leaf of Lauraceae Juss., 22°42'40"N, 100°57'28"E, alt. 1345 m, 03 October 2022, Hong Yu bis (YHH 2210035 – preserved in the Yunnan Herbal Herbarium).

##### Description.

***Sexual morph***: External mycelia produced from all orifices and sutures, often covering the host body, initially white turning brown. Stromata single to multiple, produced from dorsal pronotum, part branched, 16–24 mm in length, cylindrical, pale white to light brown, becoming pinkish at the apical part. Fertile region of lateral cushions, 1–3, commonly 2 per stroma, hemispherical, chocolate brown at maturity, 1–1.9 × 0.8–1.3 mm. Perithecia immersed to partially erumpent, flask-shaped, (211–) 218–298 (–305) × (94–) 99–142 (–158) μm, with short, exposed neck or ostiole. Asci 8-spored, hyaline, cylindrical, (92–) 96–132 (–134) × 7–11 (–13) μm. Ascus caps slightly prominent, hemispherical, 4–5 × (2–) 3–4 μm. Ascospores hyaline, thin-walled, lanceolate, tortuous arrangement in the ascus, 47–64 × 5–7 μm, 6–7-septate, gently curved at round apex, tapered end shorter than round apex.

***Asexual morph***: *Hirsutella*-A type associated with the apical part of stromata. *Hirsutella*-C type produced from the leg and antennal joints. Phialides lageniform, 54–99 μm long, 4–6 μm width at base, tapering to a long neck, 1–2 μm in width. Conidia fusiform to limoniform, 6–8 × 3–5 μm, slightly narrowing at the top.

***Germination process***: The released ascospores germinated within 48 h to produce 1–2 long and extremely narrow hair-like capilliconidiophores, (27–) 44–65 (–69) × 1–2 μm, bearing a single terminal capilliconidium, (5–) 6–9 × 3–4 (–5) μm, hyaline, smooth-walled, limoniform to fusiform, slightly narrowing and curved at the top.

***Host***: *Colobopsis* sp. (Formicinae).

##### Habitat.

Subtropical monsoon evergreen broad-leaved forest. Infected *Colobopsis* sp. biting into a leaf of Lauraceae Juss., from 1.2 to 2.4 m above the ground.

##### Distribution.

China, Yunnan Province, Puer City.

##### Material examined.

China, Yunnan, Puer City, Simao District. Infected *Colobopsis* sp. biting into a leaf of Lauraceae Juss., 22°42'40"N, 100°57'28"E, alt. 1,345 m, 03 October 2022, D.X. Tang (YHH 2210003, YHH 2210004, YHH 2210005, YHH 2210006).

##### Notes.

**Figure 1.**
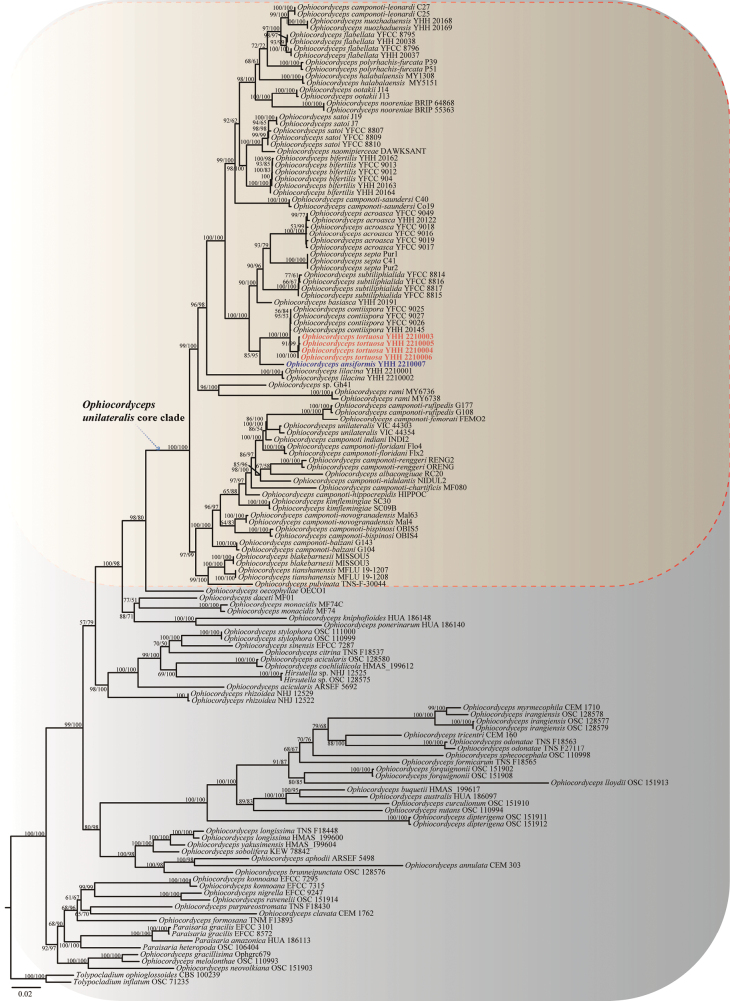
Phylogenetic tree of *Ophiocordyceps* and related genera, based on the concatenation of LSU, SSU, *TEF1α*, *RPB1* and *RPB2* sequence data. The tree was generated from an alignment of 4,827 sites and 143 taxa (38 within *O.unilateralis*). The phylogeny was inferred using the IQ-tree. Values at the nodes represent IQ-tree bootstrap proportions (on the left) and posterior probabilities (on the right). All values were shown at the nodes. The scale bar 0.02 indicates the number of expected mutations per site. The two new species were indicated in blue and red font within *O.unilateralis* core clade. Two species (*T.inflatum* OSC 71235 and *T.ophioglossoides* CBS 100239) in *Tolypocladium* were used as the outgroup taxa.

In the phylogenetic tree, the new species *O.tortuosa* was sister to *O.contiispora* (Fig. 1: BS = 100%, BPP = 100%) within *O.unilateralis* core clade (Fig. 1: BS = 100%, BPP = 100%). *Ophiocordycepstortuosa* was distinct from other species of the *O.unilateralis* core clade in that it produced lanceolate, obvious separate and tortuous arrangement ascospores in the ascus and produced branched stromata, slightly narrowing conidia (Table 3).

**Figure 2.**
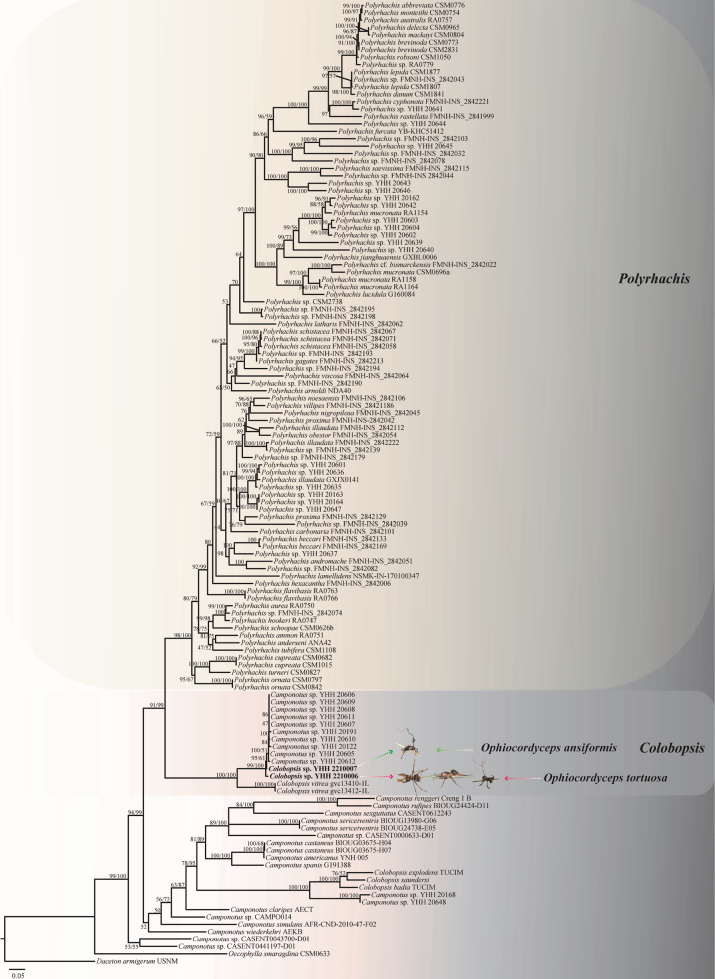
Phylogenetic tree of some genera of the Formicinae based on *COI* sequence data. The tree was generated from an alignment of 660 sites and 131 taxa. The phylogeny was inferred using the IQ-tree. Values at the nodes represent IQ-tree bootstrap proportions (on the left) and posterior probabilities (on the right). All values were shown at the nodes. The scale bar 0.05 indicates the number of expected mutations per site. The species (*Colonopsis* sp. YHH 2210006 and *Colonopsis* sp. YHH 2210007) are indicated in black and bold font in this work. The Latin name on the right of the tree refers to the pathogenic fungi infecting the host ants and the illustration refers to the fungi infecting ants in the wild. *Dacetonarmigerum* USNM was used as the outgroup taxa.

**Figure 3.**
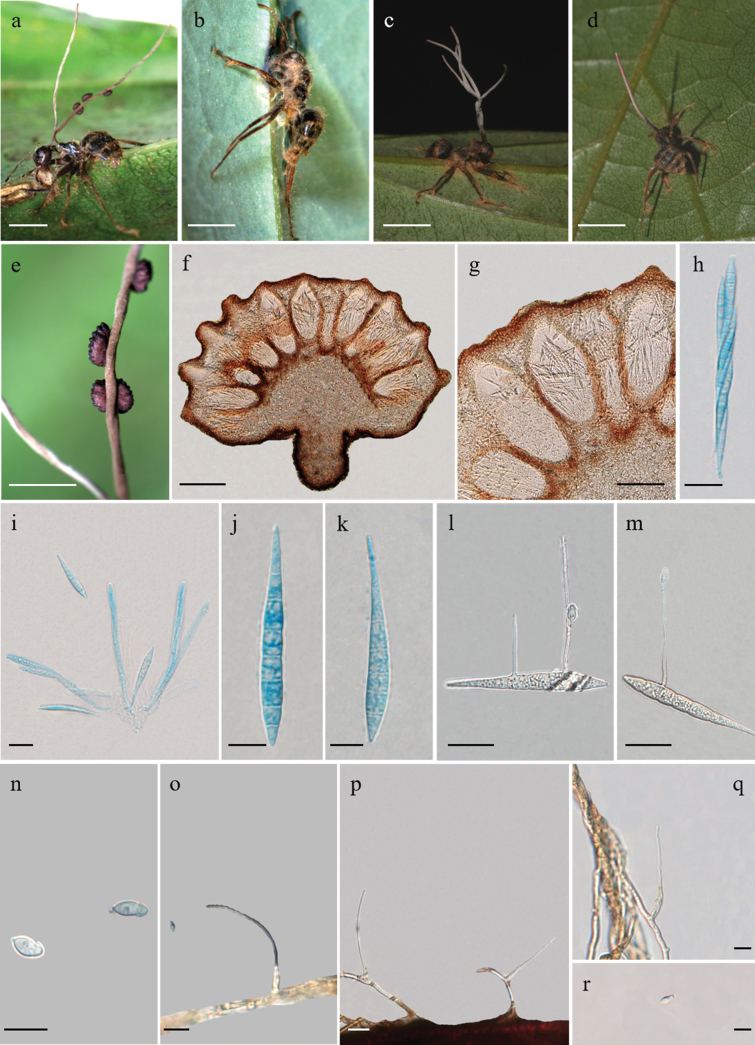
*Ophiocordycepstortuosa***a–d** infected *Colobopsis* sp. biting into a leaf of Lauraceae Juss **e** the three ascomata produced from the stroma **f, g** cross-section of ascomata showing the perithecial arrangement **h, i** asci **j, k** ascospores **l, m** ascospore with capilliconidiophores **n** capilliconidium **o–q** phialides **r** conidia. Scale bars: 4000 µm (**a, b**); 3000 µm (**c, d**); 2000 µm (**e**); 200 µm (**f**); 100 µm (**g**); 20 µm (**h, i**); 10 µm (**j, k**); 20 µm (**l, m**); 10 µm (**n**); 20 µm (**o–r**).

**Table 3.** Morphological comparison of two novel taxa and related species within *Ophiocordycepsunilateralis* complex.

Species	Host	Death position	Stromata	Ascomata	Perithecia (μm)	Asci (μm)	Prominent cap	Ascospores (μm)	Septation	Hirsutella asexual morph (μm)	Conidia (μm)	References
*Ophiocordycepsacroasca*	*Camponotus* sp.	biting leaf	single	hemispherical, 3 × 2–3 mm	ovoid, 247–296 × 176–225	cylindrical, 8-spored, 131–172 × 5–8	prominent, 3–5 × 4–6	vermiform, 83–108 × 2–3	4–5	Hirsutella-A type and Hirsutella-Ctype, 17–30 × 1–4	limoniform, 2–3 × 1–2	Tang et al. (2023b)
*Ophiocordycepsbasiasca*	*Camponotus* sp.	biting leaf	single	spherical, 3 × 2 mm	flask-shaped or ovoid, 202–242 × 102–149	cylindrical, 8-spored, 96–188 × 4–9	hemispherical, 3–5 × 4–5	vermiform, 89–119 × 2–3	4–5	Hirsutella-A type, 10–23 × 1–5	oviform, 1–4 × 1–2	Tang et al. (2023b)
*Ophiocordycepscontiispora*	*Camponotus* sp.	biting leaf	single	disc-shaped, 0.7–1 mm	flask-shaped, 158–212 × 69–122	cylindrical, 8-spored, 89–130 × 4–9	hemispherical or square, 1–3 × 3–5	fusiform, 38–48 × 2–4	no obvious separation	Hirsutella-C type, 57–92 × 1–4	olivary or flask-shaped, 4–6 × 1–2	Tang et al. (2023b)
*Ophiocordycepsansiformis*	*Colobopsis* sp.	biting leaf	single	hemispherical, 1–1.3 × 0.7–1 mm	flask-shaped, 174–290 × 99–128	cylindrical, 88–112 × 7–11	hemispherical, 4–7 × 2–4	lanceolate, 45–59 × 5–7	6–9	Hirsutella-A type, 15–24 × 3–4		This study
*Ophiocordycepssepta*	*Camponotus* sp.	biting leaf	single	hemispherical, 2 mm	fusoid-ellipsoid, 280–300 × 100–150	cylindrical, 8-spored, 125–165 × 12.5–15	–	lanceolate, 45–50 × 6–8	7–8	Hisutella-A type, 25 × 2–3; Hisutella-C type, 50 × 5.5	fusiform, 5–6 × 1–2; fusiform to narrowly lemoniform, 9 × 5	Kobmoo et al. (2015)
*Ophiocordycepssubtiliphialida*	*Camponotus* sp.	biting leaf	single	disc-shaped, 2 × 1.2–1.9 mm	flask-shaped, 195–296 × 87–161	cylindrical, 8-spored, 89–119 × 5–9	hemispherical, 2–4 × 5–7	lanceolate, 52–72 × 5–8	6–7	Hirsutella-C type, 70–116 × 1–3	olivary, 6–10 × 3–6	Tang et al. (2023b)
*Ophiocordycepstortuosa*	*Colobopsis* sp.	biting leaf	single to multiple	hemispherical, 1–1.9 × 0.8–1.3 mm	flask-shaped, 211–305 × 94–158	cylindrical, 92–134 × 7–13	hemispherical, 4–5 × 2–4	lanceolate, 47–64 × 5–7	6–7	Hirsutella-A type, 54–99 × 1–6	fusiform to limoniform, 6–8 × 3–5	This study

#### ﻿ Ophiocordyceps ansiformis

Taxon classificationFungiHypocrealesOphiocordycipitaceae

Hong Yu bis, D.X. Tang & J. Zhao sp. nov.

3A4A6D1E-2E65-57CB-95E9-9470FB84BD41

849061

Fig. 4

##### Etymology.

Ansi- = handle, formis = forms, the epithet refers to ascospores having a handle-shape.

##### Diagnosis.

*Ophiocordycepsansiformis* differs from closely-related species by producing lanceolate ascospores with a structure resembling a handle-shape in the middle, while *O.contiispora* produces fusiform ascospores that do not exhibit a similar structure in the middle.

##### Holotype.

China, Yunnan Province, Jinghong City, Puwen Town. Infected *Colobopsis* sp. (Formicinae) biting into a leaf of Rubiaceae Juss., 22°31'24"N, 100°58'57"E, alt. 1,029 m, 02 October 2022, Hong Yu bis (YHH 2210036 – preserved in the Yunnan Herbal Herbarium).

##### Description.

***Sexual morph***: External mycelia produced from all orifices and sutures, brown at maturity. Stroma single, produced from dorsal pronotum, never branched, 25–28 mm in length, cylindrical, dark brown at maturity, light brown at the apical part. Fertile region of lateral cushions, 1–3, hemispherical, 1–1.3 × 0.7–1 mm. Perithecia immersed to partially erumpent, flask-shaped, (174–) 189–290 × 99–126 (–128) μm, with short, exposed neck or ostiole. Asci 8-spored, hyaline, cylindrical, (88–) 92–108 (–112) × 7–10 (–11) μm. Ascus caps prominent, hemispherical, 4–6 (–7) × 2–3 (–4) μm. Ascospores hyaline, thin-walled, lanceolate, having a handle-shape in the middle, 45–59 × 5–6 (–7) μm, 6–9-septate, tapering at apex.

**Figure 4.**
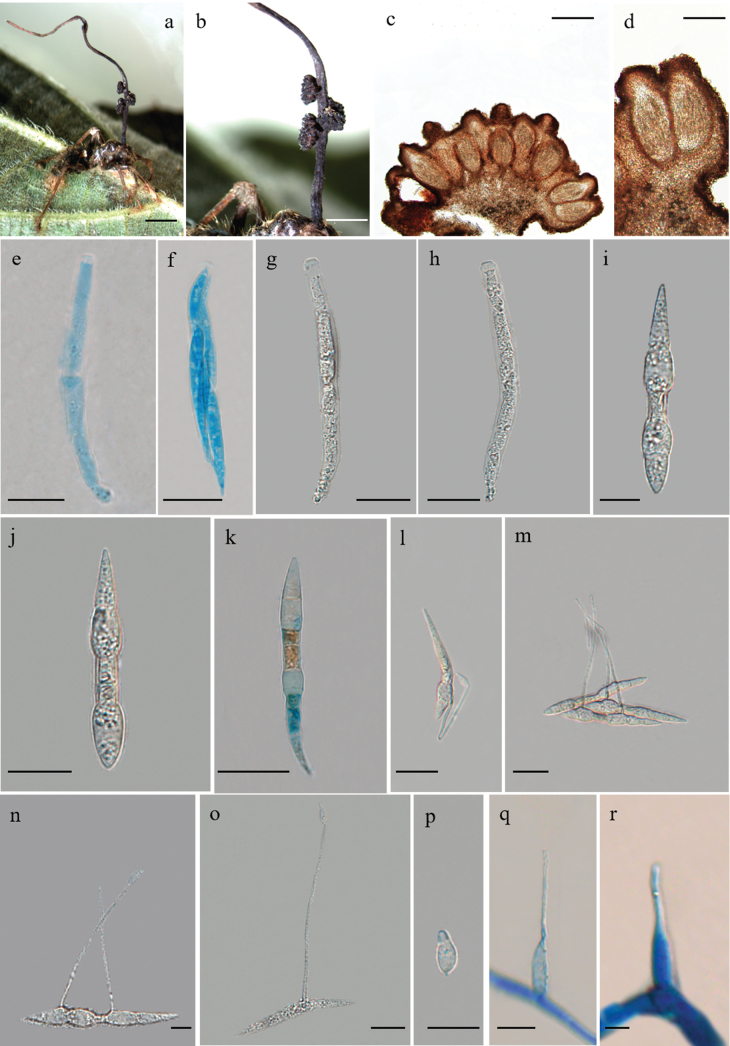
*Ophiocordycepsansiformis***a** infected *Colobopsis* sp. biting into a leaf of Rubiaceae Juss **b** the three ascomata produced from the stroma **c, d** cross-section of ascomata showing the perithecial arrangement **e–h** ascus **i–k** ascospores **l–o** ascospores with capilliconidiophores **p** capilliconidium **q** phialides. Scale bars: 4000 µm (**a**); 2000 µm (**b**); 200 µm (**c**); 100 µm (**d**); 20 µm (**e–h**); 10 µm (**i, j**); 20 µm (**k–o**); 10 µm (**p, q**); 5 µm (**r**).

***Asexual morph***: *Hirsutella*-A type present along stromata. Phialides lageniform, 15–24 × 3–4 μm, tapering to a short neck, 6–8 μm in length. Conidia were not observed.

***Germination process***: Ascospores released on agar germinated after 48 h to produce 1–2 capilliconidiophores, (54–) 60–79 (–84) × 0.8–1.4 μm, bearing a terminal capilliconidium, hyaline, smooth-walled, limoniform, 6–10 × 3–4 μm, slightly narrowing apically.

***Host***: *Colobopsis* sp. (Formicinae).

##### Habitat.

Subtropical monsoon evergreen broad-leaved forest. Infected *Colobopsis* sp. biting into a leaf of Rubiaceae Juss., from 0.8 to 1 m above the ground.

##### Distribution.

China, Yunnan Province, Jinghong City.

##### Material examined.

China, Yunnan Province, Jinghong City, Puwen Town. Infected *Colobopsis* sp. biting into a leaf of Rubiaceae Juss., 22°31'24"N, 100°58'57"E, alt. 1,029 m, 02 October 2022, D.X. Tang (YHH 2210007).

##### Notes.

Phylogenetic analyses showed that *O.ansiformis* formed a sister lineage with *O.tortuosa* and *O.contiispora*, was clustered in the *O.unilateralis* core clade, with statistical support from bootstrap proportions (BS = 85%) and Bayesian posterior probabilities (BPP = 95%) (Fig. 1). *Ophiocordycepsansiformis* was similar to *O.tortuosa* and *O.contiispora* in the same host *Colobopsis* sp. (Fig. 2). However, it differed from *O.tortuosa* and *O.contiispora* in that it produced lanceolate ascospores and has a handle-shape in the middle (Table 3).

### ﻿Key to two novel taxa and related species within *Ophiocordycepsunilateralis* complex

**Table:** 

1a	Stromata never branched	**2**
1b	Stromata part branched	***Ophiocordycepstortuosa***
2a	Ascomata hemispherical	**3**
2b	Ascomata disc-shaped	**4**
2c	Ascomata spherical	***Ophiocordycepsbasiasca***
3a	Perithecia ovoid, ascospores vermiform, 83–108 × 2–3 µm	***Ophiocordycepsacroasca***
3b	Perithecia flask-shaped, ascospores lanceolate, 45–59 × 5–7 µm	***Ophiocordycepsansiformis***
3c	Perithecia fusoid-ellipsoid, ascospores lanceolate, 45–50 × 6–8	***Ophiocordycepssepta***
4a	Ascospores fusiform, 38–48 × 2–4 µm	***Ophiocordycepscontiispora***
4b	Ascospores lanceolate, 52–72 × 5–8 µm	***Ophiocordycepssubtiliphialida***

## ﻿Discussion

Many closely-related species parasitising Hymenoptera are considered cryptic species within the genus *Ophiocordyceps*. These species are distinguished by morphological features and molecular phylogenetic studies. Known examples of these fungi were found occurring on adult ants in the *O.myrmecophila* species complex, for example, *O.megacuculla* and *O.granospora* (Khonsanit et al. 2018). Over ten species were parasitic on termites, for example, *O.asiatica*, *O.khokpasiensis*, *O.mosingtoensis*, *O.pseudorhizoidea* and *O.termiticola* (Tasanathai et al. 2019), *O.radiciformis*, *O.isopterorum*, *O.globosa*, *O.fusiformis* (Tasanathai et al. 2022), *O.puluongensis* (Xu et al. 2022) and *O.ovatospora* (Tang et al. 2022). Other groups such as *Ophiocordycepsnutans* species complex attack stink bugs (Friedrich et al. 2018; Khao-ngam et al. 2021) and *Ophiocordycepspseudoacicularis* species complex were found occurring on Lepidoptera larvae (Tasanathai et al. 2020). These complexes have been proposed as distinct species, based on molecular phylogenetic studies and morphological characteristics.

In this study, two new species, namely *O.tortuosa* and *O.ansiformis*, were established within *Ophiocordyceps*, based on a combination of morphological features, phylogenetic analyses (LSU, SSU, *TEF1a*, *RPB1* and *RPB2*) and ecological data. The *O.unilateralis* complex species was sister to *O.oecophyllae* and both are sister to the *O.kniphofioides* sub-clade. The species within *O.unilateralis* clade infects exclusively Camponotini ants (e.g. *Camponotus*, *Polyrhachis*, *Colobopsis*, *Dinomyrmex*) (Evans et al. 2018). Entomopathogenic fungi in the *O.unilateralis* complex occurring on the host ants *Camponotus* show host specificity (Araújo et al. 2018; Kobmoo et al. 2019; Lin et al. 2020; Tang et al. 2023b). However, pathogenic fungi infecting *Polyrhachis* ants do not exhibit species specificity, for example, more than nine species of *Polyrhachis* ants infected by *O.satoi* (Tang et al. 2023a) and two species of *Polyrhachis* ants (Polyrhachiscf.hookeri and *Polyrhachislydiae*) infected by *O.nooreniae* (Crous et al. 2016). The same ant host, *Camponotus* sp. (YHH 20606, 20609, 20608, 20611, 20607, 20191, 20610, 20122, 20605 and 20612), was infected by ant pathogenic fungi, including *O.basiasca*, *O.contiispora*, *O.acroasca*, *O.subtiliphialida*, *O.tortuosa* and *O.ansiformis* (Tang et al. 2023b). The ants, *Camponotus* sp. and *Colonopsis* sp. (Fig. 2), may be the same ant, separated only by a small genetic distance, based on *COI* molecular phylogenetic studies. The Tang et al. (2023b) result showed that the ant pathogenic fungi parasitising the genus *Camponotus* have host specificity and Kobmoo et al. (2019) indicated that more than one ant pathogenic species might parasitise the same host species. Based on a population genomics study, our results in this work fully prove and support the basis of the above research.

The two novel species within the *O.unilateralis* core clade showed slightly micro-morphological characteristics (the shape of ascospore, secondary germination) that made them recognised from other species. *Ophiocordycepansiformis* differed from *O.contiispora* by producing lanceolate ascospores with a handle-shape in the middle, while *O.tortuosa* differed from *O.contiispora* by producing lanceolate ascospore with obvious separation and tortuous arrangement in the ascus. In addition, *O.tortuosa* and *O.ansiformis* differed in the size of perithecia (211–305 × 94–158 µm vs. 174–290 × 99–128 µm), asci (92–134 × 7–13 µm vs. 88–112 × 7–11 µm) and ascospores (47–64 × 5–7 µm, 6–7-separate vs. 45–59 × 5–7 µm, 6–9-separate), this work supporting the idea of cryptic species (Evans et al. 2011b; Khonsanit et al. 2018; Tasanathai et al. 2019). The species in the *O.unilateralis* complex commonly bite and attach themselves on to spines, leaves, saplings, epiphytes, moss and twigs in a “death grip”, dying in an elevated position, from 0.25 m to 2 m (Evans et al. 2011b; Hughes et al. 2011; Kepler et al. 2011; Luangsa-ard et al. 2011; Kobmoo et al. 2012; Araújo et al. 2015; Crous et al. 2016; Araújo et al. 2018; Evans et al. 2018; Tang et al. 2023a, 2023b). The two new species *O.ansiformis* were biting and attached in a leaf of Rubiaceae Juss., while *O.tortuosa* was biting in a leaf of Lauraceae Juss., dying in an elevated position (*O.ansiformis* 0.8 to 1 m vs. *O.tortuosa* 1.2 to 2.4 m). We conducted a two-year tracking survey on one of the *O.unilateralis* complex species at the same location and season in subtropical monsoon evergreen broad-leaved forest. It was found that the death location of the host ant on the underside of leaves above the ground seems to be influenced by environmental factors such as rainfall, humidity, temperature etc. We found that there were differences in the location of death from the ground in the *O.unilateralis* complex at different years in the same species. In addition, Loreto et al. (2018) have shown that environmental conditions affect the biting type of host ants.

Therefore, future studies are recommended to examine the impact of changes in environmental conditions on the height at which host ants die.

We had inferred the phylogeny, based on each single gene and also used a concatenated dataset in this study. *Ophiocordycepstortuosa* was recovered sister to *O.contiispora* with strong support and consistent topology, based on the concatenated and single gene (*TEF1a* and *RPB1*) tree. The species *O.ansiformis* was also recovered sister to *O.tortuosa* + *O.contiispora* with weak to strong support and consistent topology, based on the concatenated and single gene (*TEF1a* and *RPB1*) tree (Suppl. materials 2, 3). The sister relationship between *O.tortuosa* and *O.lilacina* was recovered, based on single gene SSU tree. Sequences of *Ophiocordycepsansiformis*, *O.subtiliphialida*, *O.contiispora* and *O.basiasca* clustered together into a clade, based on the SSU tree. Single gene SSU trees were different from the topological structure of other trees (concatenated, *TEF1a* and *RPB1* tree) for the two species proposed in this work. Other species (*O.camponoti-femorati*, *O.camponoti-rufipedis* and *O.unilateralis*), based on SSU, *TEF1a* and *RPB1* trees have similar topological structures in this work. SSU phylogenies indicate their utility as a well marker to infer phylogenetic relationships at the subclass level (Tang et al. 2006), but it may be difficult to distinguish cryptic species only using the single gene SSU. To sum up, the two new species, *O.tortuosa* and *O.ansiformis*, proposed in this study, were fully supported by morphological features, phylogenetic analyses (concatenated, *TEF1a* and *RPB1* tree) and ecological data.

## Supplementary Material

XML Treatment for Ophiocordyceps tortuosa

XML Treatment for Ophiocordyceps ansiformis

## ﻿Additional information

### Author ORCIDs

Dexiang Tang https://orcid.org/0000-0002-7662-224X

Jing Zhao https://orcid.org/0000-0001-7871-2209

Yingling Lu https://orcid.org/0009-0008-8119-1975

Zhiqin Wang https://orcid.org/0000-0001-9022-3635

Tao Sun https://orcid.org/0000-0001-7837-2101

Zuoheng Liu https://orcid.org/0000-0003-4118-3694

Hong Yu https://orcid.org/0000-0002-2149-5714

### Data availability

All of the data that support the findings of this study are available in the main text or Supplementary Information.

## Supplementary materials

Supplementary material 1 Phylogenetic tree of *Ophiocordyceps* and related genera, based on single gene SSU sequence Data type tif Explanation note The phylogeny was inferred using the IQ-tree. Values at the nodes represent IQ-tree bootstrap proportions. The scale bar 0.05 indicates the number of expected mutations per site. The two new species were indicated in bold. Two species (*T.inflatum* OSC 71235 and *T.ophioglossoides* CBS 100239) in *Tolypocladium* were used as the outgroup taxa. https://binary.pensoft.net/file/922070This dataset is made available under the Open Database License (http://opendatacommons.org/licenses/odbl/1.0/). The Open Database License (ODbL) is a license agreement intended to allow users to freely share, modify, and use this Dataset while maintaining this same freedom for others, provided that the original source and author(s) are credited.Dexiang Tang, Jing Zhao, Yingling Lu, Zhiqin Wang, Tao Sun, Zuoheng Liu, Hong Yu

Supplementary material 2 Phylogenetic tree of *Ophiocordyceps* and related genera, based on single gene *TEF1a* sequence Data type tif Explanation note The phylogeny was inferred using IQ-tree. Values at the nodes represent IQ-tree bootstrap proportions. The scale bar 0.05 indicates the number of expected mutations per site. The two new species were indicated in bold. Two species (*T.inflatum* OSC 71235 and *T.ophioglossoides* CBS 100239) in *Tolypocladium* were used as the outgroup taxa. https://binary.pensoft.net/file/922071This dataset is made available under the Open Database License (http://opendatacommons.org/licenses/odbl/1.0/). The Open Database License (ODbL) is a license agreement intended to allow users to freely share, modify, and use this Dataset while maintaining this same freedom for others, provided that the original source and author(s) are credited.Dexiang Tang, Jing Zhao, Yingling Lu, Zhiqin Wang, Tao Sun, Zuoheng Liu, Hong Yu

Supplementary material 3 Phylogenetic tree of *Ophiocordyceps* and related genera, based on single gene *RPB1* sequence Data type tif Explanation note The phylogeny was inferred using the IQ-tree. Values at the nodes represent IQ-tree bootstrap proportions. The scale bar 0.05 indicates the number of expected mutations per site. The two new species were indicated in bold. Two species (*T.inflatum* OSC 71235 and *T.ophioglossoides* CBS 100239) in *Tolypocladium* were used as the outgroup taxa. https://binary.pensoft.net/file/922072This dataset is made available under the Open Database License (http://opendatacommons.org/licenses/odbl/1.0/). The Open Database License (ODbL) is a license agreement intended to allow users to freely share, modify, and use this Dataset while maintaining this same freedom for others, provided that the original source and author(s) are credited.Dexiang Tang, Jing Zhao, Yingling Lu, Zhiqin Wang, Tao Sun, Zuoheng Liu, Hong Yu
